# Khmer Girls in Action and healing justice: Expanding understandings of anti-Asian racism and public health solutions

**DOI:** 10.3389/fpubh.2022.956308

**Published:** 2022-12-20

**Authors:** May Lin

**Affiliations:** Department of Asian and Asian American Studies, Cal State Long Beach, Long Beach, CA, United States

**Keywords:** youth organizing, community organizing, Southeast Asian American, Khmer and Cambodian Americans, young women, health equity, anti-Asian racism, healing justice

## Abstract

This community case study highlights how Khmer Girls in Action (KGA), a Southeast Asian young women-led organizing group in Long Beach, California, enacts healing justice. Healing justice is a framework for both transforming structures at the crux of health inequities and healing emotional, spiritual, and psychological wounds inflicted by structural violence. KGA also anchors the cross-racial and intersectional Invest in Youth (IIY-LB) coalition. IIY-LB youth leaders have successfully fought to increase the city's investments in the social determinants of health, especially young people's well-being. Meanwhile, the coalition has critiqued over-investments in criminalization and policing as devastating Black, Brown, queer, low-income, immigrant, and refugee youth and communities. This case study highlights how KGA's work expands understandings of both anti-Asian racism and public health solutions in the following ways: First, KGA cultivates youth leaders' critical analyses to define root causes of health inequities impacting Southeast Asian refugees as rooted in imperialism, disinvestment, and increased criminalization. Furthermore, youth leaders come to understand how their communities' struggles and liberation necessitate intersectional and cross-racial coalitions. Second, youth leaders forge public health solutions that involve divesting from criminalization and institutionalizing an Office of Youth Development, as co-created with young people. Third, KGA and other IIY-LB organizations cultivate youth's leadership skills and community's political power to move hearts and minds of decision-makers and community members. For example, youth leaders have passed a ballot measure funding youth, climate, and health programs in addition to the city-based Office of Youth Development. Fourth, KGA engages in a wide range of “inward” healing practices to salve wounds caused by intergenerational trauma. This case study contributes to Asian American health equity by highlighting the specific importance of organizing, while illuminating abolitionist perspectives on public health solutions- both of which are under-discussed in discourse about anti-Asian racism. KGA's work thus illustrates the importance of centering critical analyses and leadership of communities most impacted by structural violence in forging transformative public health solutions to anti-Asian racism.

## Introduction

Asian American communities face health inequities rooted in structural violence, but obscured by the enduring model minority myth (1). As Saw and colleagues have argued, Asian American health equity requires addressing specific and heterogeneous forms of structural violence, such as “displacement, colonization, and imperialism.”^1^ Thus, health interventions must take place on multiple levels: from structural policy changes such as disaggregated data, to community and individual-level interventions, such as community-based, culturally appropriate healing.

Youth and community organizing groups are uniquely poised to address these multiple levels of health inequities: they build the leadership of those most impacted by structural violence to critically analyze, devise solutions, advocate for, and enact systemic change. Such groups have won more equitable policies around the social determinants of health, even when their efforts are not explicitly framed around health (3). Yet organizing is under-examined in public health—especially when discussing Asian Americans, who have been racialized and pitted against other communities of color as politically apathetic (4).

Uplifting Southeast Asian American (SEAA) youth organizing expands understandings of both anti-Asian racism and public health solutions. Youth organizing groups develop critical analyses of root causes of systemic inequities to create and fight for transformative solutions (5). Uplifting analyses of SEAA communities who have experienced and resisted heightened criminalization is essential, since Asian Americans are starkly polarized in how they experience and define anti-Asian racism, as well as perceive increased policing as a policy response (6). Furthermore, youth organizing can promote health on multiple levels: youth lead campaigns shaping more equitable institutions, and participants are empowered in ways that buffer negative health consequences of discrimination (7). Considering systemic barriers to civic engagement for Asian Americans (8) and increasing discrimination negatively impacting health Asian American youth (9, 10), youth organizing can promote health equity in multiple ways.

This community case study makes these connections by highlighting the healing justice work (2018–2021) of Khmer Girls in Action (KGA), a SEAA young women-led organization. KGA also anchors the cross-racial, intersectional Invest in Youth-Long Beach coalition (IIY-LB). Healing justice^2^ involves both “outward” and “inward” healing: the former focuses on transforming institutions at the crux of health inequities, while the latter involves psychological, spiritual, and emotional “healing from the wounds inflicted from structural oppression” (11). KGA develops youth leaders' understandings of, and solutions to, structural forces threatening well-being. Youth leaders build civic and political power to win resources for health equity, such as an Office of Youth Development. Finally, KGA specifically attends to emotional well-being and intergenerational healing *via* diverse practices of self and collective care. This case study encourages public health practitioners to more explicitly center and support SEAA young women and those most impacted by structural violence in creating public health solutions.

## Contexts: Long Beach and Khmer Girls in Action (KGA)

Long Beach, California is comprised of 72% communities of color (12); approximately 4.8% are Filipino, and 4% are Cambodians (13). Importantly, Long Beach is home to the largest population of Cambodians outside of Cambodia (14). KGA was founded in 1997 to focus on Cambodian young women's reproductive health and empowerment. Since then, the organization has expanded its scope as a community organizing group working toward “a safe, healthy and just world where all people are free from oppression and are able to determine their lives and communities” (15). KGA also centers an intersectional analysis around gender, class, race, sexuality, and culture.

KGA works to heal intergenerational and compounded trauma. Youth leaders come from families and communities that fled horrific genocide and mass U.S. bombings, only to resettle in disinvested areas lacking supports for healthy integration (9, 16–21). In addition to policies engineering high poverty levels and barriers to basic needs (14, 22, 23), increasingly punitive policies have converged to increase criminalization and surveillance of SEAA, along with Black and other Brown communities (16, 24–27). Subsequently, deportations have disproportionately devastated Cambodians and other SEAA groups^3^; oftentimes, women bear the brunt of ensuing financial consequences, emotional burdens, and compounded trauma (29).

KGA engages in healing justice to promote healing on individual, community, and structural levels. Their research^4^ found that Cambodian youth in Long Beach experience high levels of depression and lack educational support and reproductive health services, while battling racial profiling and deportations (9, 17). This research, along with lived experience and analyses of how structural violence fuels PTSD, intergenerational trauma, and myriad physical and mental health issues in their communities (9, 22, 30, 31), inspired youth leaders to successfully fight for a Wellness Center providing health education, preventive care, and support services (32).

KGA also works toward a more “progressive and sustainable Long Beach” *via* cross-racial coalitions addressing interlinked contexts of structural racism. Long Beach has a large “racial generation gap” wherein 86% of young people are people of color compared to 47% of seniors (12). This results in an older, whiter tax base that is reluctant to invest in public resources, such as education, that benefits youth of color (33). Yet youth of color also disproportionately experience poverty (34). KGA and other youth organizations have worked together to address these contexts within an infrastructure of coalitional organizing largely funded by The California Endowment's Building Healthy Communities Initiative. Prior to IIY-LB, youth groups worked together to address racially discriminatory punitive school discipline and push for resources for a more supportive school climate (35).

## Key programmatic details: KGA and the invest in youth campaign

KGA's program areas involve leadership development (building analysis and skills for youth to enact change); cultural and media arts; and individualized support for personal well-being and academic and career success. As first years and sophomores in high school, participants join programs separated by gender. They analyze histories and root causes of structural violence that impact their lives and begin developing leadership skills. As juniors and seniors, youth continue in co-ed leadership programs where they plan events and lead campaigns. For example, I volunteered for the “Khmer Justice Program,” where youth leaders strategize and lead campaign work, mentor younger youth, and serve as ambassadors to different coalitions.^5^

KGA anchors the Invest in Youth coalition, involving 8 other organizations.^6^ IIY-LB found that the city allocated only $204 per youth on positive youth development compared to $10,500 per youth arrest in Fiscal Year 2018 (34). The coalition has critiqued the city's over-investment in policing and incarceration and fought for more investments in positive youth development.^7^ From 2017–2018, youth leaders collected 757 surveys finding city residents prioritize mental health, youth employment, and parks and after school programs, rather than police (36).^8^ Youth leaders met regularly with city councilmembers, had their research filed as official city research, testified at city council, and planned public events. In 2018, they successfully convinced City Council to allocate funds for a youth-led strategic planning process to identify priorities for a future youth fund and office. The campaign also fought to ensure that this fund would be housed in the health department, which would have the infrastructure necessary to distribute funds.

From 2019-2020, KGA consulted on the process. IIY-LB youth leaders successfully ensured that low-income, queer, youth of color, and youth with disabilities from all city council districts would be substantively represented as youth ambassadors (YAs) developing the Youth and Emerging Adults Strategic Plan. YAs led data collection and analysis,^9^ and IIY-LB youth successfully advocated for prioritizing health and well-being in the plan. Meanwhile, leaders continued meeting with elected officials and conducting public events. KGA's Integrated Voter Engagement (IVE) program educated and mobilized voters- especially Khmer/Southeast Asian communities, youth, and low-income communities of color who experience systemic barriers to voting. The IVE program helped the coalition secure 57% of the vote for Measure US: a 15-cent tax on local oil companies funding programs for climate resilience, a Long Beach Youth Fund, and health programs. Along with other organizing efforts, IIY-LB secured City Council's approval of the strategic plan and allocation of Measure US funds toward establishing an Office of Youth Development.

## Methodology

This case study draws from a subset of my larger, community-engaged ethnographic project.^10^ This specific study analyzes 200 h of participant observation and 11 interviews^11^ that I conducted with KGA staff and youth leaders from 2018–2019 and 2021, as well as my content analysis of 75 sources including city planning documents, reports, op-eds, local newspaper and independent media articles, and social media posts. Minors under 18 signed assent forms and parents signed consent forms; adults signed consent forms. Quotations from public documents (e.g., op-eds) are attributed to real names, whereas quotes from interviewees are labeled with pseudonym initials to protect privacy. Participant observation included weekly Khmer Justice Program and Summer Organizing Institute meetings, IIY-LB meetings led by KGA youth and staff, youth-planned events, voter canvassing, and city council meetings. Semi-structured interviews addressed questions such as: impacts and lessons of KGA participation; leadership skills developed; healing justice practices; self and collective care, and reflections on campaigns. Using both inductive and deductive coding, I coded secondary sources, field notes, and/or event and interview transcriptions *via* NVivo. Codes relevant to this particular study include: healing justice, self-care, collective care, support systems, definitions of racism, and well-being. For this study, I further refined sub-themes such as “anti-Asian racism,” “coalition-building,” “health framing,” and “political education.”

## Results: How KGA redefines anti-Asian racism and promotes health equity

### Political education: Identifying root causes of anti-Asian violence and health inequities

KGA facilitates political education where youth leaders identify root causes of intergenerational trauma and structural violence as experienced and resisted by SEAA refugee communities and as connected to other communities of color. These inform nuanced understandings of anti-Asian racism.

For example, I sat in on a workshop where the organizer led youth leaders through a timeline of the “U.S. Migration-to-School-to-Deportation pipeline.” They pointed out that the timeline began decades prior to the Khmer Rouge, illustrating lineages of imperialism leading up to U.S.' dropping millions of tons of bombs in Cambodia, Vietnam, and Laos. Yet resettlement did not bring full relief: Jenn explains how contexts failed to support refugees and discusses how and why many young people's parents are pushed to low-wage labor that compounds grave tolls on their health. They then discuss policies such as welfare reform, the three strikes law, and Illegal Immigration Reform & Immigrant Responsibility Act, which have fueled criminalization and deportations of more than 750 Cambodian, 200 Laotian, and 550 Vietnamese community members as of 2018 (37). Youth leaders discuss the impacts of heartbreaking family separations and how women bear the burden of knitting together fragmented families. After all, 1/3 of respondents in KGA's 2011 study reported that someone they knew has faced deportation (17). To conclude, Jenn encourages members to reflect on how they stand on the shoulders of ancestors and their visions for collective thriving.

KGA staff and youth leaders identify these forms of structural violence as anti-Asian racism. As KGA leader Alexis wrote in the *Long Beach Post*, many Cambodian refugees facing deportation “didn't have the resources to cope with intergenerational trauma or to help their parents with translation or employment” (38). In public comment urging the City Council to support funding for positive youth development, youth leader Khyloe explained that: “When my parents came as refugees, they didn't know English or how to get food. They struggled to find work or even get income. They worked in low paying industries like garment factories and donut shops. Many in their generation had a hard time making ends meet, so they turned to robberies and selling drugs and joining gangs.” Youth leaders publicly shared stories linking the personal and political, explaining how structural violence manifests in mind, body, and soul, from grappling with heart disease and diabetes, to cycles of heartbreak and displacement.

These analyses of anti-Asian violence expand mainstream discourses of anti-Asian hate focused on specific incidents usually targeting East Asians. KGA released a statement expressing outrage and grief after the 2021 murders of Asian women in Atlanta. They connected gendered and racialized anti-Asian violence to Biden's deportation of thirty-three Vietnamese community members- even as Biden condemned anti-Asian hate. KGA pointed out that these events are part of a “a pattern of white supremacy to uphold dehumanizing systems of oppression.” Their statement urged intersectional and coalitional efforts that address insidious and everyday forms of structural violence manifesting in their community's struggles around “school, housing, employment, family separations” (39).

This statement also speaks to KGA's definitions of anti-Asian racism as intertwined with histories and futures of Black, Latinx, and Indigenous communities. Their perspective points to policing and incarceration as a form of anti-Asian racism. KGA youth leaders' previous research found that 39% of young Cambodian men surveyed had been stopped by law enforcement (17). This lived experience contrasts with that of some East Asian American groups perceiving police positively and as an appropriate solution to anti-Asian racism (6). In contrast, organizing director and KGA alum Jenn explained in a podcast interview:

“As children of refugees, we came into a system that has a history of harming Black communities and incarcerated Black and Brown youth at higher rate. And when the 1.5 refugee generation came and resettled here, they got pulled into that. We were vulnerable; we were criminalized for being poor, and our families have not even settled here for even 45 years. But what we've learned is that this all points to a bigger collective problem, and it's a call for a collective solution to address root causes” (40).

Jenn's argument is illustrative of KGA's coalitional, racial justice work. KGA leaders stated in interviews that participating in IIY-LB and previous coalitions fostered their deep personal and political connections with other communities of color. Some leaders had participated in a retreat in San Diego where they learned about and connected their own experiences to the dehumanization of Mexican migrants by a massive Border Patrol presence. Furthermore, KGA holds events such as the Feast of Resistance to help members dismantle myths about Thanksgiving and to understand settler colonialism and Native survivance. Thus, KGA's analysis of anti-Asian racism is thoroughly situated within political education highlighting connections with other communities of color.

### Defining expansive public health solutions within investment in positive youth development and resources

These nuanced analyses feed into development of transformative solutions for outward healing. Youth leaders leveraged action research and storytelling in public testimonies, op-eds, and events. They argued that over-investments in criminalization harmed their well-being and advocated for more city-level investments supporting their communities' holistic health. These explicit critiques of incarceration resonate with the American Public Health Association's assertion that abolition of police and prisons is a critical strategy for health equity (41). As KGA's executive director Lian pointed out during a press conference, the city budget is a sign that “Long Beach has historically underinvested in the health and well-being of young people.” KGA youth leader Angelina reflected that workshops where youth leaders analyzed the city budget showed how institutions would “rather punish us than give us the opportunities to heal or improve ourselves.” Such supports, as youth leaders argued in public statements, could include programs such mental health, youth leadership, and job development.

Convergences of the COVID-19 pandemic, Summer 2020 uprisings around Black lives, and backlashes to the uprising further highlighted the need for explicit analyses around abolition as public health. Indeed, the issue came to a head during the strategic planning process. City staff attempted to create a priority around “public safety” and proposed partnerships with Long Beach Police. Youth leaders were taken aback, pointing out that youth participants had never stated this as their priority. Rather, they pointed out that their well-being was harmed by police. As Jenn summarized: youth of color “are in heavily policed neighborhoods, which also leads to more fear, paranoia, mistrust, depression, and this feeling of surveillance” (40).

Instead, youth leaders asserted that the appropriate solutions necessitated investing in public health, meeting basic needs, and centering racial, gender, and socioeconomic equity. They successfully ensured that the plan's language foregrounded priorities around youth's well-being. Youth leaders' research and analyses fed into key priorities that included: physical health, mental health, and emotional wellness; planning for the future; community care, housing, and transportation. For example, the plan states a commitment to “holistic approaches to well-being, whereby youth are mentally, physically and spiritually healthy and live in safe, economically sound environments that support their overall well-being” (35). Such framing reflects IIY-LB youth leaders' recognition of investment in health, well-being, and social determinants of health as a key solution- not just to anti-Asian racism, but to structural violence impacting Long Beach's youth.

### Developing Southeast Asian youth's civic and political power–Transforming institutions that cause harm

KGA cultivates leadership skills of young people to enact “outwardly oriented” healing justice to create more equitable institutions. Youth leaders move the hearts and minds of decision-makers, peers, family, and community members through arenas such as electoral organizing. Politicians are more responsive to “likely voters,” (currently more white, affluent, facing less health conditions) who also tend to reject more equitable policies such as Medicaid expansion (42). Yet SEAA and other communities of color have historically been disenfranchised and face specific barriers to civic and political engagement. In an IIY-LB meeting, KGA leader Chelsea reflected: “my parents are refugees from the Khmer Rouge and so they have a lot of trauma around voting and being involved in politics, so they don't really want to get involved.” In 2016, only 37% of Southeast Asians registered voters in Long Beach turned out to vote (43). Turning SEAA and other disenfranchised communities into “likely voters” is an under-examined health equity strategy.

KGA's workshops empower youth to understand and educate others about the importance of electoral engagement as one of many strategies for transformative change. As Joy Yanga, KGA's communications director stated in the *Press-Telegram*, SEAA youth and youth of color can help family members and peers understand the “benefits that come with voting and having political power in the community” (44). Youth leader Alexis explained in an interview that KGA's workshops dissected various ballot initiatives and rationale for voting in different ways.

Youth also canvass, phone bank, and lobby legislators around statewide propositions and local campaigns such as Measure US (previously discussed in Programmatic Details). Especially critical is the support that young people receive from each other and adult staff. Before canvassing, organizers help young people develop their personal stories as connected to the ballot initiative. Leaders practice the script multiple times with each other and staff to become comfortable with and tailor the script and practice public speaking. Youth leaders give each other feedback and discuss ways to improve the script. Younger members are paired with older members and are always accompanied by an adult, so they are fully supported while talking with residents.

Consequently, KGA youth have influenced family, peers, and community members and fueled concrete wins for health equity. Alexis shared that her parents “will come to me about what prop[ositions] to vote for,” and that voting has become a family matter. KGA's electoral organizing fits into a longer-term strategy of building relationships with community members. Youth leaders canvass in their own neighborhoods and leverage their existing relationships. The IVE program contacts residents regularly and connects them to vital services; thus, residents grow to understand and enthusiastically support KGA's work. As such, KGA was able to win voters' support of Measure US; they also convinced over 700 residents to voice their support for using Measure US for the Office of Youth Development and other health programs.

KGA youth leaders also plan, perform in, and emcee creative public events to engage the broader public and conduct delegation meetings to win over key decision-makers. Events include a wellness week featuring various health and wellness activities and an annual “Haunted House” around Halloween highlighting different campaigns. Jenn argued that this format helps to “personify the horrors” of structural violence that their campaigns address and “highlight the mental health aspect of… the true impact of these ballot measures” (44). Youth members participate in different planning committees led by staff members, such as art, logistics, and media outreach, to implement an artistic vision in these events.

Staff also support youth to link their personal stories to the issues they advocate for and to tell their stories in compelling ways. Youth leader Emily reflected in an interview on preparing to emcee an event:

[KGA staff] sat down with me and [the other emcee] to create our scripts. He told us, “Be yourself, you don't have to be so professional, be more expressive.” He gave us some tips on how to really project our voices and have fun with it. We edited [the script] to make it sound like our own. I feel [supported] with my co-emcee and [staff] saying, “you got this girl, you have the script if anything goes wrong; you can do this.”

Emily's story reflected youth leaders' interviews where they shared that they always had ample time where staff encouraged them to practices and personalize their scripts. As Angelina stated, “it's very empowering because we don't see a lot of youth speaking out and [being] given a space to practice.” These efforts thus illustrate how KGA's organizing equips young people to lead outwards aspects of healing justice, which also provides individual benefits for well-being.

### Self and collective care and inwardly oriented healing practices

Much of KGA's “outward” healing is interlinked to inward healing. For example, Alexis shared that KGA provided legal and community support to prevent her uncle's deportation; previous research shows that deportations takes grave tolls on deportees' and their families' health (45), while family reunification is linked to positive health outcomes (46). However, organizing for systemic change can be exhausting, potentially even compounding trauma and negative health consequences (47). KGA staff shared in interviews that taking time to heal is especially critical given contexts of intergenerational trauma and ongoing systemic violence. Youth leader Chelsea reflected, “We deserve love and happiness just as much as [social and] economic policies we [are] fighting for with KGA”: signaling the importance of attending to individual well-being.

As such, KGA engages in a diverse range of self and collective care practices, including arts, culture, and other practices that involve the senses and, as Lian described, “interrupt our cellular memory of trauma.” For example, youth members often create visual art, and their annual arts showcase, Yellow Lounge, involves youth learning, creating, and performing classical Khmer dance, poetry, Theater of the Oppressed, hip-hop dance, and more. Recuperating these cultural practices is a critical form of healing that resist multiple layers of systemic erasure and invisibilization.

Furthermore, as Lian shared in an interview, KGA intentionally builds in time for play and rest: whether incorporating fun icebreakers, breathing, meditation, and tai chi practices into retreats and weekly meetings, or dedicating a day each month to wellness activities. Wellness days have included movie-watching, workshops herbal teas or remedies, or trips to the aquarium or beach. As Chelsea reflected:

“When we have a relaxation day where all we do is watch a movie, talk about our feelings and sit in a circle- that's enough self-care for me to get through whatever BS I'm going through in high school. That sanctuary space is needed… because I don't know what I would've done [otherwise].”

In interviews, KGA leaders shared that they feel holistically supported. KGA provides food and transportation, as well as supportive relationships essential to their well-being. Staff and youth have regular check-ins, which Alexis shared:

“Really helped me talk about my problems. It was really good for me to talk to someone about it because… I would keep it quiet [before]. But [KGA staff] were very dedicated in having a sit-down talk with me when I needed it… Joining KGA made me realize that mental health is very important, physical health is very important. The stuff that I go through at home is not something that I'm going through by myself. I don't have to keep it to myself. I can reach out for help.”

She also shared that members develop a “buddy system” to regularly check in on and support each other. Many youth leaders also shared in interviews that KGA's academic and career support program- especially the college application support–alleviated academic stress. For example, Emily reflected that KGA's belief in her leadership and academic support helped her continue in the program while juggling school, extracurricular, and caregiving responsibilities.

KGA also provides a unique space for young people to process intergenerational trauma and specific, intersectional experiences of being SEAA young women, including intersections with queer and non-binary identities. As Emily shared, KGA helped her “embrace my sexuality,” and connected her to queer youth of color spaces while encouraging her to lead workshops on queer identity. Meanwhile, Chelsea explained that these shared experiences helped her “find comfort in staff and [members] who were really supportive and often going through the same thing.” Many young people shared that KGA helped them heal relationships with their parents. As Alexis stated:

“They really help us learn to communicate how we feel with our parents. I feel a lot closer to my mom because of KGA….I did tell her about the work that we're doing, and she was really inspired by it… She will come to some of [our] events.”

KGA helped young people understand what their parents had endured and survived, while encouraging youth to pursue their own interests and passions. Chelsea reflected on how KGA mentors helped her “to find my voice in art,” which helped her feel less pressure about school because “I just feel comfortable in what I'm doing in my life.” KGA staff supported her to navigate college and financial aid applications and mediated a conversation with her parents to win their support for pursuing her dream school and major.

Finally, engaging in leadership and organizing activities boosts psychological empowerment, pride in identity, and other positive impacts on health (7). Young people see the real outcomes of their organizing, which supports their hopefulness and sense of empowerment. Chelsea explained that “KGA created a space for me to come into power, I didn't realize how much youth have been involved in local policies, and there are ways for us to have our voice heard.” Similarly, Alexis reflected that participating in this campaign “showed what a difference I can make,” and nurtured her optimism that they would win “more in the future.” As such, the inward and outward aspects of healing justice can also reinforce each other.

## Conclusion and summary

Khmer Girls in Action illuminates how healing justice- that is, engaging in inwardly oriented healing while building the leadership and power of SEAA young women, in coalition with other youth and communities of color- has advanced health equity. Centering perspectives of SEAA young women expanded definitions of, and solutions to, anti-Asian racism. Their critical understandings of policing, incarceration, criminalization, and deportations targeting Cambodian and other SEAA communities illustrate connections to other communities of color. However, KGA and other organizing groups grapple with the need for more sustained, general funding to engage in long-term work needed for healing structural violence. The following recommendations suggest how public health practitioners can more actively center and support these efforts.

### Ensure substantive inclusion of SEAA communities and organizing groups in public health- community partnerships

Previous scholarship has rightfully pointed to the importance of public health partnerships with Asian American community organizations, such as service providers and community-based healing (1, 48). KGA's work points to how organizing is distinct in grappling with power and supporting those most impacted by health inequity to shift institutions. Furthermore, structural violence faced by SEAA communities fosters distinct perspectives less frequently foregrounded in Asian American discourse. Public health practitioners should create explicit guidelines to require inclusion of SEAA and other Asian American groups (e.g., South Asians) and organizing groups in public health- community partnerships.

### Cultivate long-term listening around campaigns for health equity

KGA youth leaders developed their own analyses and solutions for health equity and successfully pushed the local health department and city to implement their vision. Elsewhere, public health departments could promote understandings of community-based health solutions by developing long-term relationships to listen to how organizing groups define and advocate for health solutions, rather than first creating programs and recruiting community partnerships after the fact.

### Support co-governance, not just consultation with youth organizing groups

Public health and community partnerships must go beyond consultation. KGA pushed city staff to substantively include and listen to youth voice in multiple arenas. As of 2022, youth leaders are co-creating the Office of Youth Development with the city. Public health practitioners can fund youth organizing groups to provide trainings and best practices to support substantive co-development of health equity solutions.

### Critically analyze power and politics involved in devising public health solutions

KGA's work has explicitly grappled with power. Implementing police and prison abolition as a public health strategy will further require confronting power. Public health departments can learn from youth and community organizing group's analyses of power to devise more expansive solutions. For example, public health departments can fund community organizations to lead power analyses to truly understand and address root causes of health inequities.

### Leverage public health framing and funding to support organizing work

KGA and IIY-LB leveraged public health frames to advance a positive vision of youth well-being and mobilize support. Public health departments, practitioners, and scholars can more concretely support these efforts by diverting funds to organizing groups' efforts. They could also provide data and support organizing groups' efforts to link their efforts to health equity frames (49). They could help such organizations garner more support from health-oriented foundations.

KGA's healing justice work encompasses multiple complexities- from the specificity of Southeast Asian young women's perspectives as connected to broader systems of structural violence, to contradictions and confluences between transforming systems and attending to deeply, personally felt pain. KGA enacts collective care on multiple levels to dismantle anti-Asian racism and advance public health. Centering the brilliance, leadership, creativity, full humanity, and expansive vision of those most impacted by structural violence is necessary for all of us to thrive.

## Acknowledgment of constraints

This case study is deeply rooted in lessons from Cambodian American and Southeast Asian American youth-led organizations with the specific context of Long Beach from 2018–2021. As such, replication in other contexts may vary greatly due to infrastructures of community/health organizing; funding or lack thereof; and politicians' will. Furthermore, this case study comes from perspectives focusing on KGA youth and staff, and the author's perspectives as a volunteer and supporter of KGA. Further study should involve perspectives from city officials, staff, and those who are not directly involved in KGA or its coalitional efforts. Asian Americans are a heterogeneous community and far from united on policing and incarceration as solutions or root causes of anti-Asian racism and health inequities. Nevertheless, this campaign highlights how Asian American public health practitioners should grapple with the politics and power involved in public health rather than assuming homogeneous political stances.

## Data availability statement

The original contributions presented in the study are included in the article/supplementary material, further inquiries can be directed to the corresponding author/s.

## Ethics statement

The studies involving human participants were reviewed and approved by University of Southern California; California State University, Long Beach. Written informed consent to participate in this study was provided by the participants' legal guardian/next of kin. Written informed consent was obtained from the individual(s) for the publication of any potentially identifiable images or data included in this article.

## Author contributions

The author confirms being the sole writer of this work, with edits and support provided by KGA staff and youth, and has approved it for publication.

## Footnotes

^1^Similarly, Hansen and Metzl argue for the need for structural and institutional-level interventions that create “health-promoting” environments, neighborhoods, and policies (2).

^2^As discussed by scholars and organizers such as Shawn Ginwright and Cara Page.

^3^Southeast Asian Americans are more likely to be deported based on old criminal convictions compared with others; and between 2018-2020, the deportation of Cambodian nationals increased by 279 percent (28).

^4^In collaboration with researchers from UCLA's Social Work School.

^5^As with other youth organizing groups, staff center youth decision-making and voice as much as possible in all aspects- for example, in deciding tactics for campaigns, campaign directions, theme of events, artistic vision for events, the content of events, and more.

^6^Organizations serve and/or are led by low-income Black, Latinx, queer, and/or gender non-conforming youth. Other organizations include: Californians for Justice; Gender and Sexualities Alliance Network; California Conference for Equality and Justice; Educated Men with Meaningful Messages; Success in Challenges/Children's Defense Fund-California, Filipino Migrant Center, and Advancement Project. Other foci include: Black & Latinx children; youth of color statewide; Southeast Asian men; Filipino communities; and youth, educator, and allies. The organizations also use different approaches- some focus on organizing, others on direct services and advocacy.

^7^As defined within the strategic plan: “engagement of youth along with their families, communities, and/or governments so that youth are empowered to reach their full potential. PYD approaches builds skills, assets, and competencies; foster healthy relationships; strengthen the environment; and transform systems” (35).

^8^Action research led by youth in collaboration with Cal State Long Beach. Cal State Long Beach professors and grad students from the Department of Geography led the development of research questions and IRB approval. Youth, with the support of KGA staff, took the lead on collecting surveys through events discussed above.

^9^This included: strategic planning trainings; facilitated community forums (including responding to 2020 uprisings for Black liberation); designed and conducted interviews; co-designed survey instruments; collected survey data from 787 youth; and analyzed findings.

^10^Initially approved by University of Southern California's IRB, with follow-up research approved by Cal State Long Beach's IRB.

^11^Interviews more generally came from snowball sampling. This includes 6 interviews with KGA staff and 6 with youth leaders.
